# The risk factors of nonunion after intramedullary nailing fixation of femur shaft fracture in middle age patients

**DOI:** 10.1097/MD.0000000000016559

**Published:** 2019-07-19

**Authors:** Kuan-Jou Wu, Shu-Hao Li, Kuang-Ting Yeh, Ing-Ho Chen, Ru-Ping Lee, Tzai-Chiu Yu, Cheng-Huan Peng, Kuan-Lin Liu, Ting-Kuo Yao, Jen-Hung Wang, Wen-Tien Wu

**Affiliations:** a School of Medicine, Tzu Chi University; b Department of Orthopedics, Hualien Tzu Chi Hospital, Buddhist Tzu Chi Medical Foundation; c Institute of Medical Sciences, Tzu Chi University; d Department of Research, Hualien Tzu Chi Hospital, Buddhist Tzu Chi Medical Foundation, Hualien, Taiwan.

**Keywords:** femur shaft fracture, intramedullary nail fixation, oligotrophic non-union, RUST score

## Abstract

Although the optimal treatment for femur shaft fracture is intramedullary nailing fixation, nonunion still occurs. We determined the oligotrophic nonunion rate among femur fractures managed operatively and identified risk factors for reoperation. This was a retrospective clinical study. The data of the patients between 40 and 70 years old with diaphyseal femur fracture who have received reamed and interlocked intramedullary nailing fixation in our hospital from February 2014 to April 2018 were collected. They were followed at regular intervals for at least 1 year after the operation. The primary outcome was nonunion of the fracture site that required reoperation in accordance with the radiographic union scale for tibial shaft fracture (RUST), which is a reasonable score system for lower limb diaphyseal fracture. Three of them were hypertrophic nonunion (1.9%) and the other 13 cases were oligotrophic nonunion (8.6%) at postoperative 12 months follow-up. All of the postoperative plain films showed adequate reduction quality. The three hypertrophic nonunion cases were all obese male with fracture site comminution. Fracture at the proximal third junction, hypertension (HTN) and diabetes mellitus (DM) was significantly associated with oligotrophic nonunion of the fracture site from logistic regression analysis. The mean RUST score 3 months after the operation was not significantly different between the union group and nonunion group but was significantly lower 6 months after the operation in the nonunion group. In conclusion, intramedullary nailing of the femur shaft fractures was associated with a low risk of nonunion at the 1-year follow-up in the middle age group. Those with comorbidity such as obese, HTN, and DM, with fracture site at the proximal third junction or comminution should be followed up closely and rehabilitation with cause aggressively. Radiographic scale as the RUST score at postoperative 6 months could be used to predict this complication.

## 1 Introduction

The femur plays a vital role in weight bearing in the lower extremities. The incidence of femur shaft fractures is 10 to 37 per 100,000 patients annually, with a peak in young men at age 27 years and in older women at age 80 years.^[1]^ High energy forces such as those that occur in car accidents and falling are common causes of femur shaft fracture. The common classification system for femur shaft fracture is the AO-Müller/Orthopaedic Trauma Association (AO/OTA) system, which includes the classification of all long bone fractures and femur shafts (diaphyseal), with fracture patterns mainly divided into three types: simple, wedge, and complex fractures.^[2]^ Intramedullary nailing (IMN) is the first choice for managing femur shaft fracture with low complication rates (4.9%) and an excellent option for aseptic nonunions of noncomminuted femoral shaft fractures with union rates reported to range from 72% to 100%.^[3,4]^ However, nonunion still challenges orthopaedic surgeons and poses considerable socioeconomic challenges for patients due to the required reoperations, prolonged morbidity, and inability to return to normal activity.^[5]^ Open fractures, tobacco use, delayed weight bearing, comminution of the fracture site, instability of fracture reduction, nonsteroidal anti-inflammatory drugs may intervene in fracture healing and inadequate choice of nail diameter were reported as the risk factors of nonunion of femur shaft fracture.^[6,7]^ Weresh et al revealed that age and the nail locking technique were identified as being very strongly associated to propensity of healing.^[8]^ Therefore, it is critical to understand fracture union in middle age patients because this will lead to positive clinical outcomes and early back to work. The aim of this study was to prove the hypothesis that the related union scale at 3 or 6 months after the operation may predict the future nonunion and to evaluate the risk factors for nonunion after the IMN of femur shaft fractures in middle age patient group.

## 2 Materials and methods

This retrospective study was approved by the Institutional Review Board of Hualien Tzu Chi Hospital, Buddhist Tzu Chi Medical Foundation. Patients with femur shaft fracture, either unilaterally or bilaterally, who received close reduction and internal fixation with IMN in Hualien Tzu Chi Hospital between February 2014 and December 2016 were enrolled in the study. Those who had 32-A1 to 32-B3 type fractures according to the AO/OTA classification^[9]^ and who received IMN with regular postoperative follow-up for at least 9 months were included. Patients were excluded if they had pathological fractures, a previous injury over the affected femur shaft, or primary treatment with a plate or external fixator. Those who lacked radiographic imaging of the femur fracture or complete demographic data were also excluded. Demographic data—namely age; sex; body mass index; past underlying medical diseases such as hypertension (HTN), diabetes mellitus (DM), and malignancies; duration of hospitalisation after the operation; and the kind of painkillers used after the operation—were collected. The BMI was calculated as the weight (kg) divided by the square of the height (m) and was categorized according to the World Health Organization (WHO) classification, with cut-off points of <18.49 kg/m^2^ (underweight), 18.50 to 24.99 kg/m^2^ (normal weight), and ≧25.00 to 29.99 kg/m^2^ (overweight and obese).^[10]^ So, we treat it as one of our analytic result. We have added related reference in this section.

The operative method of IMN for these patients was closed reduction with fracture-table traction under intraoperative fluoroscopic guidance and fixation with a locking nail system (Aesculap, Tuttlingen, Germany). All the nails in the study were reamed and interlocked nails. All of the surgeons have made great efforts to obtain a tight isthmal fit. Full weight bearing and quadriceps muscle training was begun on postoperative day 3. Complete partial-weight-bearing exercises with the assistance of crutches starting 6 weeks after the operation and to complete full-weight-bearing exercises since 3 months after the operation.

There is no standard method for evaluating radiographic union scale for femur shaft fracture, so we used the radiographic union scale for tibial fractures (RUST) developed by Whelan et al to evaluate the healing status of femur shaft fracture at postoperative 3 and 6 months, which is a reasonable assessment method for the lower limb diaphyseal fractures.^[11–13]^ The assessment examines callus formation and the visibility of the fracture line in the anterior, posterior, lateral, and medial cortex on anteroposterior (AP) and lateral radiographs. The medial and lateral cortices on an AP radiograph and the anterior and posterior cortices on a lateral radiograph were each given a numerical score of 1, 2, or 3 based on the following:

1. absence of a callus and visible fracture line in the cortex;
2. presence of a callus with a visible fracture line between the bridging callus; and
3. callus present with no visible fracture line in the bridging callus.

The RUST score is the sum of the scores for each cortex and thus ranges from 4 to 12. Preoperative, postoperative, and follow-up radiographic images were taken in the outpatient department 3, 6, 9, and 12 months after the IMN surgery, and all images were interpreted by three doctors. The definition of nonunion included both radiographic and clinical parts. The radiographic part was defined as when no visible progressive signs of healing were identified for at least 3 consecutive months, when the fractured bone had not healed within 9 months, or if the orthopaedic surgeon considered the fracture to have little or no chance of healing.^[7]^ The clinical part was defined as persistent pain and soreness feeling of the thigh during walk.

Statistical analyses were performed using SPSS Statistics 17.0 (SPSS Inc., Chicago, IL, USA). Comparisons between the union group and nonunion group were analysed using an independent *t* test for continuous variables and a chi-square test for categorical variables. The risk factors for nonunion were analysed using the multiple regression method. All differences were considered statistically significant at *P* < .05 and the definition of marginal significance is that .05 < *P* < .1.^[14]^

## 3 Results

We reviewed the data of 221 cases of femur shaft fracture receiving IMN technique and 69 of them were excluded due to loss of further regularly orthopedic outpatient department follow-up during the 9 months after the operation. Overall, 152 patients were included in this study, 16 of which received a diagnosis of nonunion and thus subsequently received IMN. Three of them were hypertrophic nonunion (1.9%) and the other 13 cases were oligotrophic nonunion (8.6%) at postoperative 12 months follow-up. All of the postoperative plain films showed adequate reduction quality at the immediate postoperative plain films. There were 100 males and 52 females, with a mean age of 53.2 ± 11.6 years. Overall, 82 (53.9%) of the patients were older than 50 years and 94 (61.8%) of them were overweight and obese (Table 1). Of the patients, 91 (59.9%) suffered from femur shaft fracture located in the middle third and 111 (73.0%) had 32A type fracture as per the AO/OTA classification. 131 (86.2%) of the patients were under admission for <10 days. 74 (48.7%) of the patients used NSAID for more than 1 month, while 67 (44.1%) of them received any kind of analgesics for more than 3 months after the operation (Table 1). 39 (25.7%) of the patients smoked, 42 (25.6%) regularly consumed alcohol, and 20 (13.2%) regularly chewed betel nuts. 44 (28.9%) cases had HTN and 37 (24.3%) cases had DM. The three cases of hypertrophic nonunion were all obese male and complicated with fracture site comminution (Table 1). The patients were divided into the union and nonunion groups based on the final results of revised surgery after a time point postoperative 9 months. The intraclass correlation coefficient and the interclass correlation coefficient of the RUST scores of the three doctors were 0.95 and 0.97, respectively. The mean RUST score at 3 months after the operation was not significantly different between the union and nonunion groups but was significantly lower 6 months after the operation in the nonunion group (Table 2). Logistic analysis was performed to analyse the risk factors related to oligotrophic nonunion. Univariate analysis revealed that proximal third shaft fracture, HTN, and DM significantly increased the risk of nonunion, whereas multivariate analysis revealed that proximal third shaft fracture, HTN, and DM had significant effects (Table 3). Furthermore, being overweight and obese marginally increased the risk of nonunion in the multivariate analysis.

**Table 1:**
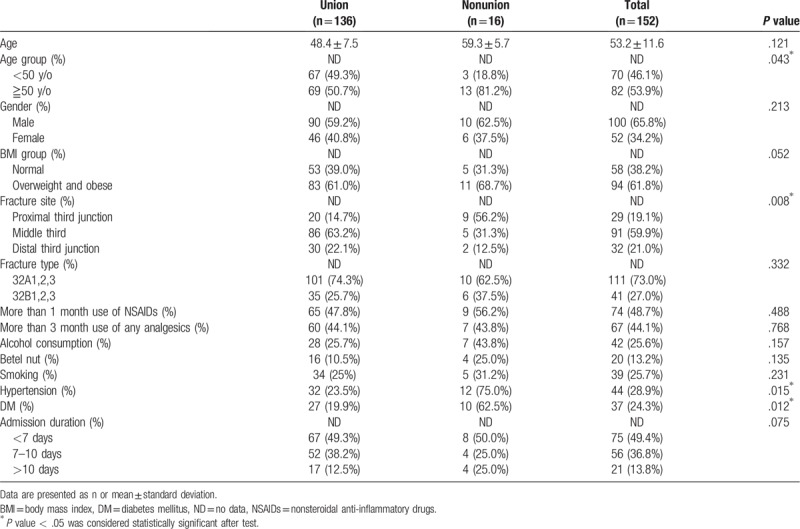
Demographic data of the patients who achieved solid union over femoral shaft Fx site at postoperative 18 months (union group) and those who were complicated with nonunion site at the time point (nonunion group) (n = 152).

**Table 2:**
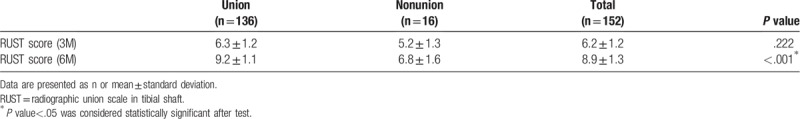
RUST Score Change Between Union and Nonunion Groups (n = 152).

**Table 3:**
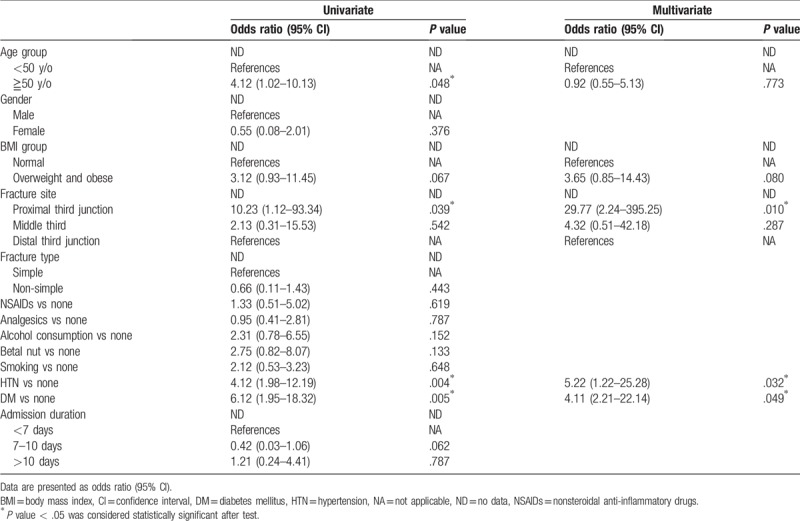
Risk factors associated with oligotrophic nonunion (n = 152).

### 3.1 Case presentation

**Figure 1:**
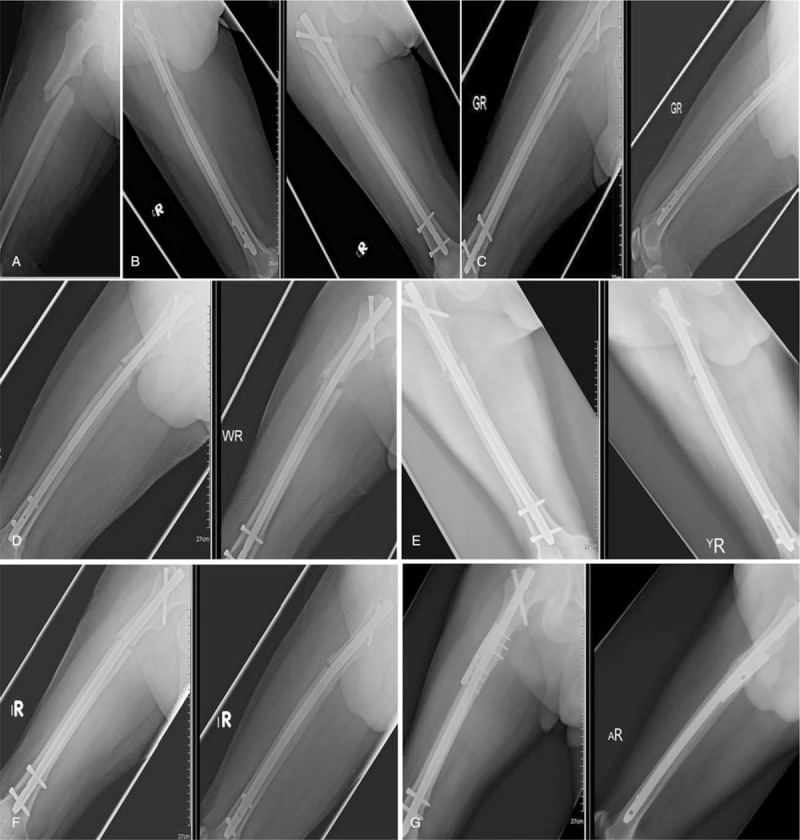
51 y/o obese man suffered from traffic accident. (A) Immediate X-ray after trauma showed right femur shaft fracture at proximal to third junction; (B) postoperative 1 day X-ray; (C) postoperative 3 months X-ray; (D) postoperative 6 months X-ray showed oligotrophic nonunion of the fracture site; (E) post-second-operation 1 day X-ray showed thicker nail and chipped bone graft over fracture site; (F) post-second-operation 4 months X-ray showed nail broke at the fracture site; (G) post-third-operation 8 months X-ray showed solid union of the fracture site.

This 51 y/o obese gardener with HTN suffered from traffic accident and diagnosed as right femur shaft fracture at proximal-middle third junction (Fig. 1a). Emergent close reduction under fluoroscopy with traction table with IMN technique with 11 mm (diameter) × 410 mm (length) nail was performed (Fig. 1b). The RUST score was 4 at postoperative 3 months and was 6 at postoperative 6 months (Fig. 1c and d). Oligotrophic nonunion of femur shaft fracture site was shown on the plain films. Revision ILN with 12 mm (diameter) × 410 mm (length) nail and mixed chipped bone grafting (autogenous bone from right iliac crest and artificial bone) by exposing the fracture site were performed at postoperative 10 months (Fig. 1e). However, he fell down at fourth months after the second operation and the nail was found broken (Fig. 1f). Revision ILN, autogenous bone grafting from left side iliac crest, and additional plating at anterior aspect of fracture site were performed. Solid union was noted at 8 months after the third operation (Fig. 1g).

## 4 Discussion

Closed reduction and IMN are the mainstream surgical treatments for femur shaft fracture.^[3]^ Oligotrophic nonunion of femur shaft facture remains a challenge for orthopaedic surgeons and is a serious socioeconomic problem for patients who need to return to work.^[4,5]^ Avascular change at a fracture site often results from open fracture or open reduction, which occasionally leads to oligotrophic nonunion, whereas unstable fixation contributes to hypertrophic nonunion. In this study, we included 152 middle aged patients with closed femur shaft fracture who underwent closed reduction with IMN. The nonunion rate was 10.5%. Thirteen patients experienced oligotrophic nonunion and three experienced hypertrophic nonunion. Although the risk of nonunion of femur shaft fracture remains unclear, studies have proposed influential factors such as open fracture, delayed weight bearing, tobacco use, fracture classification, distal fracture type, and DM.^[6,15]^ In this study, the proportion of patients over the age of 45 years who had fracture at the proximal third junction and a chronic illness (namely DM and HTN) was higher in the nonunion group than in the union group. Fracture at the proximal third junction as well as HTN and DM significantly increased the risk of nonunion. Closed reduction under fluoroscopy with intramedullary interlocking nail insertion is known as the most suitable method for treating middle isthmus femur shaft fracture due to the close fit between the nail and fracture site. Fracture from the proximal or distal junction to the isthmus may increase the chance of unstable fixation using the same surgical method. The progression of DM may alter the metabolism of bone and healing of the surrounding soft tissue, which may further decrease the rate of fracture healing and cause other complications, such as infection, malunion, nonunion, and reoperation of those patients who were surgically treated lower extremity fractures.^[16]^ TN has rarely been found to have a significant correlation with fracture nonunion in the previous studies. We assumed that this may have been for two reasons: frequent falls from dizziness caused by unstable blood pressure and a poor supply of nutrition due to intravascular pathologies caused by HTN. These conditions may be commoner in the middle age group than in younger group. Long-term use of NSAIDs and the severity of concomitant injuries may both have a negative effect on bone healing due to interference in the acute inflammation stage of fracture healing.^[7]^ We used the administration of analgesics for more than 3 consecutive months and admission of more than 2 weeks as risk factors representing concomitant or more severe injuries. The results revealed no significance over the three risk factors. Adequate analgesics, such as NSAIDs, acetaminophen, or narcotics, should be given according to the tolerance and needs of the patients because this may help them to rehabilitate sooner and more smoothly. Being overweight and obese appeared to increase the risk of nonunion with marginal significance, perhaps because these conditions increase the stress at the fracture site and on the implant during rehabilitation and weight bearing.

Delayed union is defined as the healing rate below the average rate of the fracture at the location and type of fracture and the period of normal union is usually known as 3 to 6 months.^[17]^ Conversely, a fracture of the shaft of long bone should not be considered to be nonunion until at least 6 months after the injury because union often requires a longer time period.^[18]^ The RUST score is a reliable radiographic method for evaluating the status of long bone fracture unions.^[11,12]^ In our study, we found that the RUST score in the nonunion group was not significantly different from that in the union group 3 months after the operation but was significantly lower than that 6 months after the operation. Therefore, nonunion may be predicted between 3 and 6 months after an operation. Aggressive radiographic follow-up and medication for accelerating bone healing may help considerably during this period.

Based on the results of this study we may consider two clinical implications related to femur shaft fracture receiving IMN technique in the middle age patients:

1. Additional fixation or protection and closely monitor the union status and the improved design of rehabilitation for those who had more comorbidities, overweight or fracture site at the proximal third shaft;
2. The lower RUST scores at 3 to 6 months after the operation may indicate for future complication as nonunion so that the earlier intervention of accelerating bone healing or constructing addition stability can be planned in the early stage.

This study had several limitations. First, we did not collect clinical parameters, which may have provided more clues for predicting nonunion in the early stages of bone healing. Second, we did not collect surgical diameters (such as the chosen nail diameter vs the canal diameter), the duration of operations, or the estimated surgical blood loss, which may have correlated with nonunion or the included risk factors. The nonunion risk is not as low as the previous study may be caused by the higher comorbidities and poorer bone quality in the middle age group than in the younger age group. However we did not usually check bone mineral density of all the patients, so we cannot find the effect of osteopenia or osteoporosis on the fracture union status, which is an important limitation of this study.^[3,4]^ Third, we did not confirm the compliance of the patients to their doctors’ orders in terms of ambulation exercises and activities, which may be a pertinent factor affecting bone growth and the quality of fixation stability during the healing stage. Future studies should consider addressing these limitations to improve the union rate of femur shaft fractures based on early intervention.

## 5 Conclusions

Nonunion of femur shaft fracture is a multifactorial process and the impacts of related risk factors may differ for different treatment modalities and patient characteristics, especially in the middle age group. According to radiological data and medical records, we found that oligotrophic nonunion in the femur shaft following nailing was significantly related to HTN, DM and the fracture site being at the proximal third junction, while the hypertrophic nonunion may be related to obese and fracture site comminution. The RUST score at 6 months after the operation may be used to predict nonunion in femur shafts following nailing.

## Acknowledgment

This manuscript was edited by Wallace Academic Editing.

## Author contributions

**Conceptualization:** Kuang-Ting Yeh, Ing-Ho Chen, Tzai-Chu Yu.

**Data curation:** Kuan-Jou Wu, Shu-Hao Li, Kuang-Ting Yeh, Cheng-Huan Peng, Kuan-Lin Liu, Ting-Kuo Yao.

**Investigation:** Kuan-Jou Wu, Shu-Hao Li.

**Methodology:** Ru-Ping Lee, Jen-Hung Wang.

**Resources:** Ing-Ho Chen, Tzai-Chu Yu, Cheng-Huan Peng, Kuan-Lin Liu, Ting-Kuo Yao.

**Software:** Jen-Hung Wang.

**Supervision:** Ru-Ping Lee, Wen-Tien Wu.

**Validation:** Wen-Tien Wu.

**Visualization:** Shu-Hao Li, Jen-Hung Wang.

**Writing – original draft:** Kuan-Jou Wu, Kuang-Ting Yeh.

**Writing – review & editing:** Wen-Tien Wu.
